# Revealing VNN1: An Emerging and Promising Target for Inflammation and Redox Balance

**DOI:** 10.1002/iid3.70274

**Published:** 2025-10-15

**Authors:** Linxi Lv, Tian Wang, Wenzhan Xie, Jialong Wei, Laixian Zhou, Xiaopei Qiu, Hui Feng, Wei Gu

**Affiliations:** ^1^ Center of Smart Laboratory and Molecular Medicine, School of Medicine Chongqing University Chongqing China

**Keywords:** immunometabolism, inflammatory diseases, ROS, targeted therapy, VNN1

## Abstract

**Introduction:**

The intricate balance between immunometabolic homeostasis and redox equilibrium is crucial for maintaining health, and its dysregulation is implicated in a wide spectrum of diseases. Vascular non‐inflammatory molecule‐1 (VNN1) is an emerging pantetheinase that sits at the crossroads of inflammation and metabolism, yet a comprehensive review that synthesizes its tissue‐ and disease‐specific roles and systematically evaluates its potential as a therapeutic target remains lacking.

**Methods:**

A systematic literature search was conducted to identify relevant domestic and international studies on VNN1. The search included databases such as PubMed using keywords related to VNN1′s structure and function, the disease roles of its metabolites (pantothenic acid, cysteamine), or inhibitors efficacy. The selected studies were critically reviewed and summarized to extract key pathways, inhibitor profiles and molecular docking analyses synthesized.

**Results:**

VNN1 hydrolyzes pantetheine to generate metabolites essential for CoA synthesis and glutathione redox balance. Its upregulation is closely associated with the pathogenesis of acute and chronic inflammatory diseases and certain cancers, often serving as a biomarker for disease severity. Inhibiting VNN1, either genetically or pharmacologically with compounds like RR6, OMP‐7, or natural products such as oleuropein, demonstrates significant anti‐inflammatory and antioxidant effects in preclinical models.

**Conclusions:**

VNN1 represents a promising therapeutic target for modulating oxidative stress and immunometabolism in various diseases. Future research should develop disease‐specific inhibitors, clarify tissue‐specific mechanisms, and conduct clinical trials for translation.

## 1 Introduction

Immunometabolic homeostasis alongside a delicate redox equilibrium are crucial for proper body functionality. Unsurprisingly, dysregulation within these processes is associated with the development of a diverse spectrum of pathological states, ranging from acute inflammatory diseases to chronic degenerative diseases or even cancers [1, 2]. Recent advances in understanding key molecules in biological events have improved pathogenesis insights and spurred therapeutic innovation [3]. Amongst them, vascular non‐inflammatory molecule‐1 (VNN1; also known as Vanin‐1), a 70 kDa GPI‐anchored protein with hydrolase activity, emerges as a nexus molecule bridging inflammation and metabolism [4, 5].

In 1996, VNN1 was characterized as a surface molecule essential for mouse bone marrow cell migration into the thymus [4]. Its expression varies between species, being detected primarily in human liver, spleen, thymus, and intestinal tissues, and in murine liver, kidney, ovary, intestine, and various epithelial tissues. Interestingly, early research of VNN1 failed to demonstrate its clear association with inflammatory diseases. Furthermore, no significant homology was observed when comparing VNN1 with any known adhesion molecule [4]. The 1998 discovery of its chromosomal locus (6q23‐q24), a hotspot for metastatic cancer drivers, and its sequence similarity to biotinidase (BTD), suggested latent metabolic functions [6]. This foreshadowing materialized in 2012 when crystallographic studies delineated VNN1′s enzymatic cleavage of pantetheine into pantothenic acid (vitamin B5 precursor) and cysteamine (potent antioxidant), directly linking its catalytic core to redox regulation and metabolic flux [7, 8]. The dual function of VNN1 as an enzymatic catalyst and a signaling molecule is supported by its X‐ray crystal structure [7].

The paradigm expanded in 2007 with the discovery of the IL‐13/sIL‐13Rα2‐VNN1 axis, redefining its immunological footprint through antigen‐presenting cells (APCs) activation and neutrophilic inflammation [9]. After this, a growing number of studies have since unveiled the intricate role of VNN1 in modulating oxidative stress, inflammatory processes, and cell motility in vivo, specifically through its regulation of gene expression [10, 11]. Research has demonstrated that VNN1 expression is significantly elevated in inflammatory enterocytes and colon cells [12]. Moreover, mice deficient in VNN1 in experimental models exhibit better control over inflammatory responses and intestinal damage [10, 13]. Thus, elevated VNN1 levels seem detrimental to intestinal health. Nevertheless, the VNN1‐pantetheinase pathway is acknowledged as a crucial regulator in the biosynthesis of short‐chain fatty acids (SCFA) as well as in the maintenance of beneficial mucosal barrier integrity [14]. Although this pathway only offers a limited degree of protection to the intestinal mucosa [14], VNN1 knocking‐down or deletion has been witnessed to attenuate the efficacy of therapeutic treatments for respiratory diseases [15]. Consequently, aberrations in VNN1 and associated pathways can give rise to complex metabolite alterations or inflammation‐related immunometabolic disorders, thereby exerting a negative impact on the body′s homeostasis.

Given that VNN1′s dual enzymatic/signaling functions are uniquely positioned at the crossroads of immunometabolism and redox regulation—a nexus underexplored in current therapeutic strategies—this review aims to synthesize fragmented evidence into a coherent framework, addressing the critical gap in understanding its context‐dependent roles across diseases. Here, we examine the pivotal role of VNN1 in immunometabolism as well as its potential implications in the pathogenesis of various diseases. Additionally, we discuss recent advancement in developing small molecule inhibitors in battling VNN1 dysfunction. It is our expectation that a thorough understanding of these processes will motivate fundamental research and provide theoretical support for the development and refinement of novel VNN1 inhibitors.

## 2 Pantetheinase and Its Metabolites

**Figure 1:**
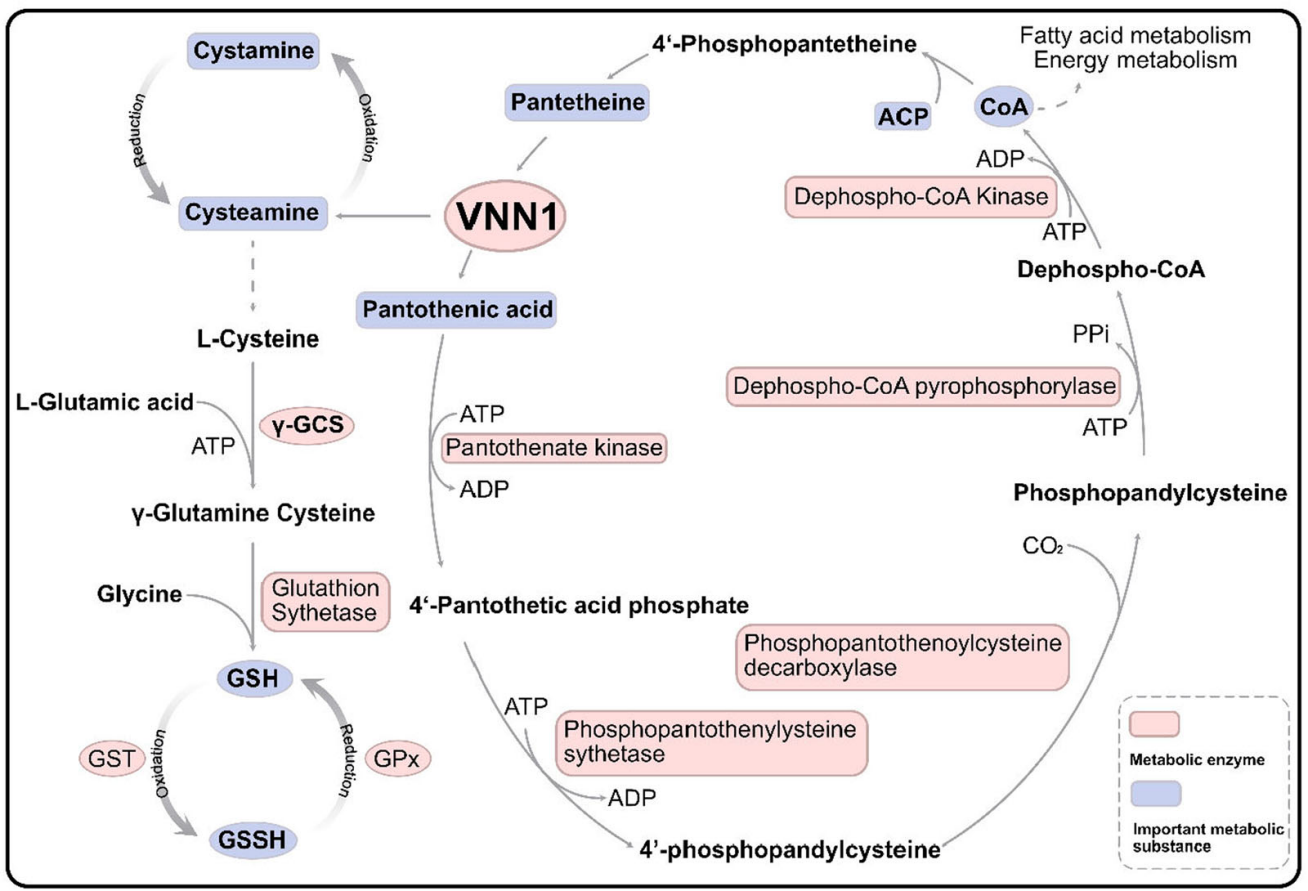
Schematic diagram of the VNN1‐pantetheinase metabolic pathway. VNN1 catalyzes the hydrolysis of pantetheine to generate pantothenic acid and cysteamine. Pantothenic acid is subsequently recycled through the CoA biosynthesis pathway, while cysteamine is metabolized to cysteine in vivo. γ‐GCS (γ‐glutamylcysteine synthetase) serves as the rate‐limiting enzyme in glutathione (GSH) biosynthesis. The redox homeostasis between reduced GSH and oxidized glutathione (GSSG) is maintained through the catalytic activities of GST (glutathione S‐transferase) and GPx (glutathione peroxidase).

The functions of VNN1 largely depend on its enzymatic activity and the corresponding metabolites [16, 17]. During the catabolism of coenzyme A (CoA), VNN1 hydrolyzes pantetheine to produce pantothenic acid and cysteamine, both of which have established antioxidant effects [18]. Pantothenic acid, an essential component of CoA, acts as a cofactor in sulfonamide and choline acetylation reactions [19]. It is also a key constituent of acyl carrier protein (ACP), facilitating the transfer of acyl groups during metabolic processes [19]. In the gastrointestinal tract, dietary CoA and ACP are first hydrolyzed by phosphatases to release pantetheine [20, 21], which is subsequently cleaved by VNN1 into pantothenate and cysteamine [22, 23]. This process enables the recycling of pantothenic acid for de novo CoA synthesis. These metabolic processes are summarized in Figure 1. The catalytic reaction scheme of VNN1 acting as a pantetheine hydrolase:

$$\text{Pantetheine}\overset{\text{VNN}1}{\to }\text{Pantothenic acid}\;+\;\text{Cysteamine}$$

### 2.1 Pantothenic Acid

Known as vitamin B5, pantothenic acid is essential for synthesizing CoA, facilitating fatty acid metabolism, and supporting overall cellular energy production. It is rarely deficient in individuals due to its abundance in various foods. However, severe malnutrition can lead to a deficiency, often accompanied by deficiencies in other nutrients [24, 25]. Vitamin B deficiency is linked to pathological mechanisms involving acetylcholine deficiency, neurodegenerative disorders, demyelination, and age‐related cognitive decline, such as Huntington′s disease (HD) [26]. Given their importance in CoA biosynthesis and lipid metabolism, vitamin B and its derivatives are often used to treat dyslipidemia. They have been shown to reduce various markers of cardiovascular disease, including low‐density lipoprotein (LDL), high‐density lipoprotein (HDL), and total cholesterol levels [27].

Following the increased focus on nonsteroidal anti‐inflammatory drugs (NSAIDs) and the use of vitamin B5 in managing skin conditions, a study on atopic dermatitis (AD) explored using a pantothenic acid analog, dexpanthenol, as an alternative therapy [28]. Dexpanthenol demonstrated superior efficacy in alleviating mild to moderate pediatric AD compared to the standard treatment with hydrocortisone. It was also effective in managing mucocutaneous side effects associated with tretinoin therapy, such as mucous membrane dryness, cheilitis, and xerosis [29]. Numerous clinical trials have assessed dexpanthenol′s efficacy in enhancing wound healing [30, 31, 32]. The findings indicated that dexpanthenol tablets and sprays promoted wound healing in postoperative scenarios like tracheal intubation, endoscopic sinus surgery, and tonsillectomy. These pieces of evidence clearly demonstrate that targeting the pantothenic acid pathway represents an effective and promising anti‐inflammatory strategy with great potential.

### 2.2 Cysteamine

Cysteamine, a thiol‐containing amine, undergoes oxidation to form the disulfide dimer cystamine. Collectively, cysteamine and cystamine serve as key regulators of oxidative stress, orchestrating thiol‐disulfide equilibrium in cellular environments through protein disulfide exchange reactions [33, 34]. At physiological concentrations, cysteamine forms a soluble complex with cysteine, facilitating its transport across cellular membranes. Once intracellular, cysteine is rapidly incorporated into glutathione (GSH) biosynthesis by γ‐glutamylcysteine synthetase (γ‐GCS), the rate‐limiting enzyme in the production of this key antioxidant. Conversely, at elevated levels, cysteamine inhibits glutathione peroxidase activity, attenuating the enzyme‐catalyzed oxidation of GSH to glutathione disulfide (GSSG) and thereby modulating intracellular redox homeostasis [35, 36, 37]. This is accompanied by an increase in the metabolic byproduct cystamine, which specifically inactivates a variety of target proteins through S‐cysteaminylation (forming covalent bonds with protein cysteine residues), including protein kinase C‐ε (PKCε), γ‐glutamylcysteine synthetase, and tissue transglutaminase [38]. This is pathologically linked to the onset and progression of neurodegenerative and inflammatory diseases, among others.

Beyond its role in redox balance, cysteamine exhibits multifaceted biological activities. It is recognized for its potent antioxidant and antimicrobial properties, as well as its ability to facilitate the conversion of cystine to cysteine—a key step in sulfur amino acid metabolism. Notably, cysteamine has been translated into clinical applications: first used therapeutically in 1976 for the treatment of cystinosis, it later received FDA approval in 1994 for managing neurodegenerative disorders such as HD and Parkinson′s disease [35, 39].

Mechanistic studies highlight cysteamine′s anti‐inflammatory potential. In cellular models exposed to lipopolysaccharides (LPS) or pro‐inflammatory cytokines, cysteamine inhibits nitric oxide (NO) production, reduces inducible nitric oxide synthase (iNOS) expression, and blocks NF‐κB activation [40, 41]. In vivo studies showed cysteamine could alleviate imiquimod‐induced inflammation in psoriatic skin by inhibiting transglutaminase 3 (TGM3) [41]. It also mitigates oxidative damage by suppressing lipid peroxidation, inhibiting protein nitrosylation, enhancing catalase activity, and scavenging reactive oxygen species (ROS) such as peroxides and superoxide radicals, thereby limiting apoptosis‐induced cell injury [42, 43]. In preclinical models of cigarette smoke/aging‐induced COPD‐emphysema, cysteamine demonstrates protective effects by reducing aggresome formation, p62 accumulation, protein poly‐ubiquitination, and apoptotic damage. Additionally, it exhibits potential efficacy against *Pseudomonas aeruginosa* infection [44]. Collectively, these findings underscore the critical role of maintaining optimal cysteamine levels in immune regulation and disease pathogenesis.

Furthermore, cysteamine shows promising antimicrobial potential, as highlighted by a study by Palucci et al. [34] showing significant efficacy against *Mycobacterium abscessus* (*M. abscessus*) [34, 45]. Granuloma‐like structures (GLS) models were constructed using *M. abscessus* rough (MAB‐R) and smooth (MAB‐S) variants. Results demonstrated cysteamine/cystamine enhanced human macrophage (THP‐1) antimicrobial activity, inhibiting bacterial growth, reducing bacterial load, abscess volume, and inflammatory response. Improved treatment outcomes were noted with amikacin [34]. Despite its effectiveness, cysteamine is not without drawbacks and may lead to adverse effects, including lupus induction risk [46]. It may also affect ocular development, causing structural abnormalities and visual dysfunction [47].

Collectively, the intricate interplay between VNN1 activity, cysteamine/cystamine dynamics, and downstream signaling pathways thus positions VNN1 as a promising therapeutic target for dissecting and modulating oxidative stress‐related disorders.

## 3 The Potential Role of VNN1 in Diseases

### 3.1 Acute Diseases

Recent studies have predominantly tended to suggest that VNN1 may serve as a disease biomarker. The majority of evidence indicates that the dynamic expression pattern of VNN1 provides novel insights for the early diagnosis of inflammatory diseases. VNN1 is upregulation in acute kidney injury (AKI) [48, 49], invasive pneumococcal disease (IPD) [50], and sepsis [51, 52] with early pathological changes, suggesting its soluble forms in urine or blood may serve as non‐invasive biomarkers. In a prospective cohort study, Lu et al. found a significant correlation between high plasma VNN1 levels in individuals experiencing trauma and an increased susceptibility to both sepsis and multiple organ dysfunction syndrome (MODS) [51]. They claimed that VNN1 showed better diagnostic performance compared to traditional biomarkers such as C‐reactive protein (CRP), procalcitonin (PCT), and Acute Physiology and Chronic Health Evaluation II (APACHE II) score.

Chen et al. [53] demonstrated that VNN1 upregulation is associated with impaired renal repair after ischemia/reperfusion (I/R) injury. Besides, in vivo experiments revealed that VNN1 knockout mice exhibit faster recovery of serum creatinine and urea nitrogen levels post‐I/R injury, along with reduced renal fibrosis and tubular cell aging [53].

Several common pro‐inflammatory factors often induce the rapid expression of VNN1 (Table 1). For instance, VNN1 expression is markedly upregulated by bacterial pathogens such as *Escherichia coli (E. coli)*, *Pseudomonas aeruginosa (Pa)*, and *Salmonella typhimurium (Sty)*, as well as their derivatives like LPS [40, 41, 54]. Remarkably, the transcript levels of *Vnn1* exhibited a substantial increase, reaching up to 400 times higher in individuals afflicted with ulcerative colitis [12].

**Table 1:** Diseases associated with the upregulation of VNN1 expression.

Disease	Host	Fold change vs. healthy control	Function	Experimental model	Ref.
**Inflammation**					
IBD	Stool from patients with UC	400	IBD biomarker	4% DSS‐induced colitis in mice	[12]
AKI	Urine from mice	∼55	AKI biomarker	BALB/c mice were intraperitoneally injected with 20 mg/kg cisplatin	[48]
Sepsis	Plasma from patients with severe trauma	> 2	Sepsis biomarker	NA	[51]
Diabetes	Plasma from obese diabetic patients	> 4.5	Lipid metabolism	NA	[55]
CKD	spot urine from adult hypertensive patients	NA	Biomarker of hypertensive renal injury	NA	[56, 57]
Bronchial asthma	IUGR mice	> 3	Promote inflammation	OVA induces asthma in IUGR BALB/c mice	[58]
Pancreatitis	*WT* and *S100A9* ^−/−^ mice; H6C7 cells	∼1.6	Protein‐protein interactions, mediate ROS release	STC induces liver injury in the AP model C57BL/6 mice and H6C7 cells	[59]
Renal I/R injury	*WT* and *VNN1* ^−/−^ mice; primary renal tubular epithelial cells	> 2	Promote cellular senescence, fibrosis, and inflammation	BALB/c mice were used to establish an I/R injury model	[53]
SSc	*WT* and *VNN1* ^−/−^ mice; primary skin fibroblasts	> 2	Anti‐fibrosis, anti‐inflammatory	BALB/c female mice were intradermally injected with HOCI to induce SSc models	[60]
**Cancer**					
AML	NA	NA	Biomarker of relapse risk and poor prognosis in AML	NA	[61]
Pancreatic cancer	Peripheral blood from patients with pancreatic cancer and diabetes	~1.4	Blood biomarker for pancreatic cancer	NA	[62]
PDA	Tissue from patients with PCAND; BxPC‐3 cells, INS‐1 cells; β‐TC‐6 cells, MIA‐PaCa‐2 cells	NA	Aggravate paraneoplastic islet dysfunction; Increased oxidative stress; PCAND biomarker	NA	[63]
Colorectal cancer	Data from patients treated with preoperative radiotherapy plus radical proctectomy	NA	Predict poor prognosis	NA	[64]
	293T cells HCT8 and HT29 cells *GPRC5A* ^−/−^ mice	> 2	Promote inflammation;	AOM + DSS induced colorectal cancer mice; DSS‐induced colitis in mice	[65]

Abbreviations: AKI, acute kidney injury; AML, acute myeloid leukemia; CKD, chronic kidney disease; I/R, ischemia/reperfusion; OVA, ovalbumin; PCAND, pancreatic cancer‐associated new‐onset diabetes; PDA, pancreatic ductal adenocarcinoma; SSc, systemic sclerosis; STC, sodium taurocholate.

**Figure 2:**
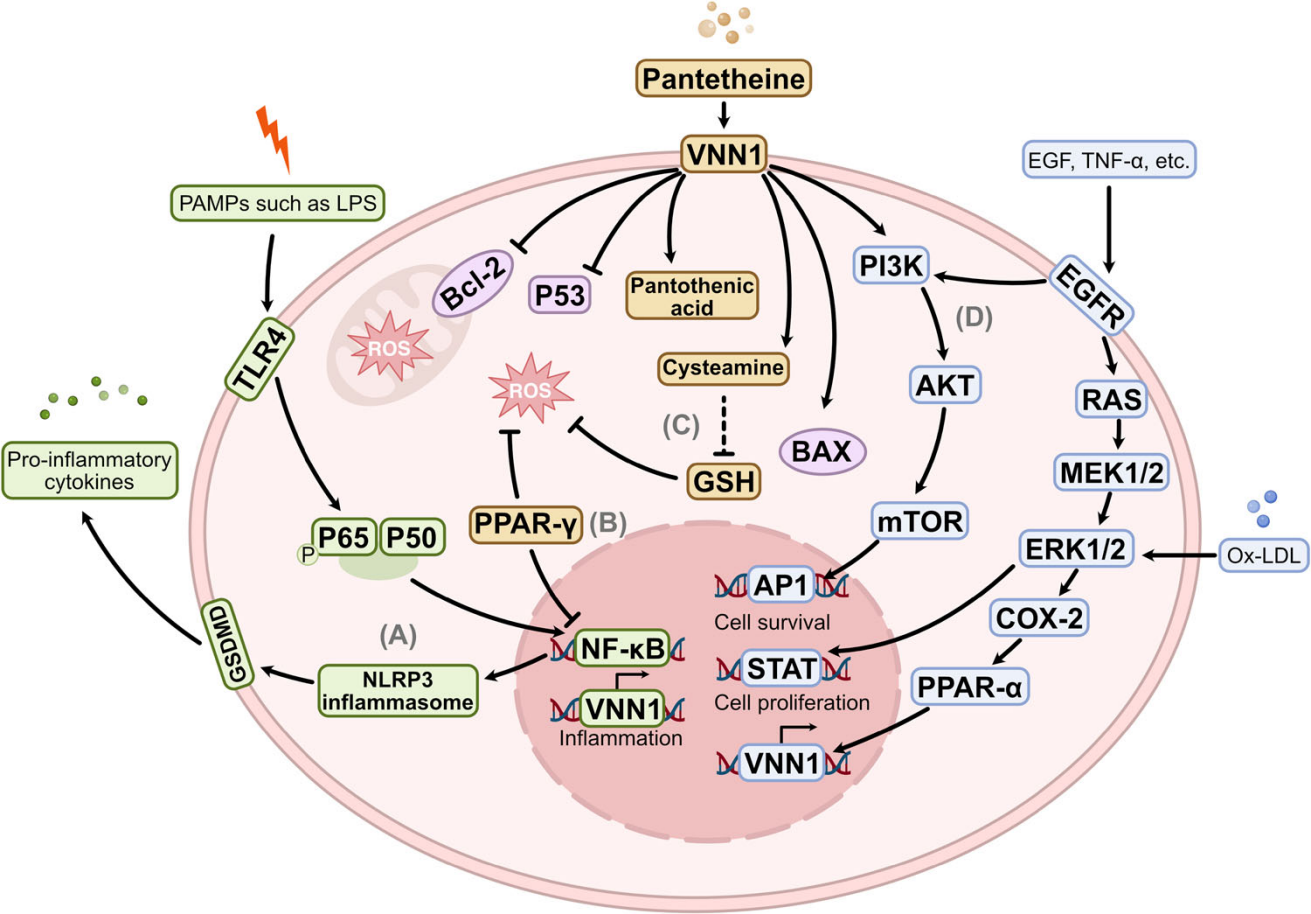
Schematic diagram of the potential role of VNN1 in inflammation. (A) Pro‐inflammatory regulation: Upregulation of VNN1 potentially promotes the activation of NF‐κB and the NLRP3 inflammasome, leading to the release of IL‐1β, IL‐6, and TNF‐α. (B) Lipid metabolism reprogramming: VNN1 affects intracellular lipid homeostasis through PPARα/γ‐dependent pathways, influencing fatty acid oxidation and lipid droplet accumulation. (C) Redox imbalance: Excessive cysteamine depletes glutathione reserves by reducing the GSH/GSSG ratio, thereby exacerbating oxidative stress. (D) Cell fate determination: VNN1 influences the balance of survival‐proliferation‐apoptosis through interactions with the PI3K/Akt and MAPK signaling cascades.

The induction of VNN1 expression involves a complex interplay of multiple signaling pathways (Figure 2). Specifically, VNN1 is modulated by cytokines including transforming growth factors (TGF) and tumor necrosis factors (TNF), and intracellular sensors like S100 calcium‐binding protein A9 (S100A9). Indeed, it is noteworthy that elevated VNN1 levels often correlate with the excessive secretion of pro‐inflammatory cytokines such as IL‐6, IL‐1β, and TNF‐α [52, 66, 67]. For instance, Xing et al. [58] found that increased VNN1 expression activates the PI3K/Akt/NF‐κB pathway, thereby inducing the production of ROS and inflammatory responses, which in turn contribute to the pathogenesis of asthma in individuals with intrauterine growth retardation (IUGR). The interaction between peroxisome proliferator‐activated receptor γ coactivator‐1α (PGC‐1α) and hepatocyte nuclear factor‐4α (HNF‐4α) also enhances VNN1 expression through facilitating the methylation of its promoter [68]. Moreover, in vivo experiments showed that VNN1 knockdown in bronchial epithelial cells reduces Akt^Ser473^ phosphorylation, ROS production, and synthesis of IL‐1β and TGF‐β [68]. Additionally, evidence indicates that S100A9 interacts with VNN1 through direct protein‐protein interactions, leading to a series of downstream effects, including the generation of ROS, activation of the NLRP3 inflammasome, and subsequent cellular damage. The primary mechanism involves the formation of a salt bridge between the Lys57 and Glu92 residues of S100A9 and VNN1 [59]. These findings suggest that VNN1 may function as a signaling molecule in inflammatory responses.

### 3.2 Chronic Diseases and Tumors

This biomarker potential extends to chronic inflammatory conditions, where elevated plasma VNN1 levels have been consistently observed in chronic obstructive pulmonary disease (COPD) [69] and non‐alcoholic steatohepatitis (NASH) [70].

Studies have demonstrated that VNN1 not only regulates oxidative stress and inflammation, but also influences intracellular glucose and lipid metabolism [8]. In research exploring the pathophysiology and vascular function associated with rheumatoid arthritis (RA) and atherosclerosis, differential expression of VNN1 was identified through a comprehensive gene analysis of peripheral blood mononuclear cells (PBMCs) from patients [71].

Another study found that oxidized low‐density lipoprotein (Ox‐LDL) modulates VNN1 expression and activity, facilitating the progression of atherosclerosis [72]. Mechanistically, Ox‐LDL upregulates VNN1 expression via the extracellular signal‐regulated kinase (ERK)−1/2/cyclooxygenase‐2 (COX‐2)/PPAR‐α signaling pathway. This upregulation impairs cellular cholesterol efflux by downregulating the expression of ATP‐binding cassette transporter A1 (ABCA1), ATP‐binding cassette G1 (ABCG1), scavenger receptor Class B Type I (SR‐BI), and Niemann‐Pick disease type C1 (NPC1) through the suppression of PPAR‐γ and liver X receptor‐alpha (LXR‐α). Additionally, VNN1 promotes inflammation by stimulating the expression of TNF‐α, IL‐1β, IL‐6, intercellular cell adhesion molecule‐1 (ICAM‐1, CD54), and vascular cell adhesion molecule‐1 (VCAM‐1). In vivo observations suggest that VNN1 may contribute to the development of atherosclerotic plaques in ApoE^−/−^ mice fed a high‐fat diet (HFD) [72]. This is evidenced by elevated triglycerides and LDL‐cholesterol levels, reduced HDL‐cholesterol levels, impaired reverse cholesterol transport, and increased inflammatory markers in plasma. In this study, they reported an interesting and uncommon phenomenon. Namely, VNN1 inhibits Ox‐LDL‐induced apoptosis in vascular smooth muscle cells by modulating p53 and Bcl‐2. Conversely, its regulatory effect is different in macrophages. This cell type‐specific regulation suggests VNN1′s multifunctional role in tissue homeostasis. Given the paucity of extant studies, further research is imperative to elucidate the relationship between aberrant VNN1 expression and cell fate.

In this context, inhibition of VNN1 expression seems beneficial in slowing disease progression due to abnormal inflammation, fibrosis, autoimmunity, and oxidative stress. Research indicates a strong association between VNN1 expression and pantothenic acid levels in both skin and plasma of systemic sclerosis (SSc) patients, closely correlating with disease severity [60]. In a mouse model of SSc induced by hypochlorous acid, VNN1 elimination effectively halted critical disease manifestations like fibrosis, immune dysregulation, and endothelial dysfunction [60].

Extending this scope to oncological context, a similar phenomenon has also been noted, where an increased expression of G protein‐coupled receptor family C Group 5 Type A (GPRC5A) in CRC tissues is shown to promote oxidative stress by regulating NF‐κB‐mediated VNN1 expression [65]. Mechanistically, GPRC5A activates the NF‐κB pathway through TNF‐α and other cytokines, leading to enhanced VNN1 expression. Higher cysteamine levels subsequently inhibit γ‐GCS activity, reduce GSH levels, and favor tumor cell proliferation. This disruption in immunometabolism and oxidative stress is closely associated with intestinal tumor development and progression.

## 4 The Existing Inhibitors

**Figure 3:**
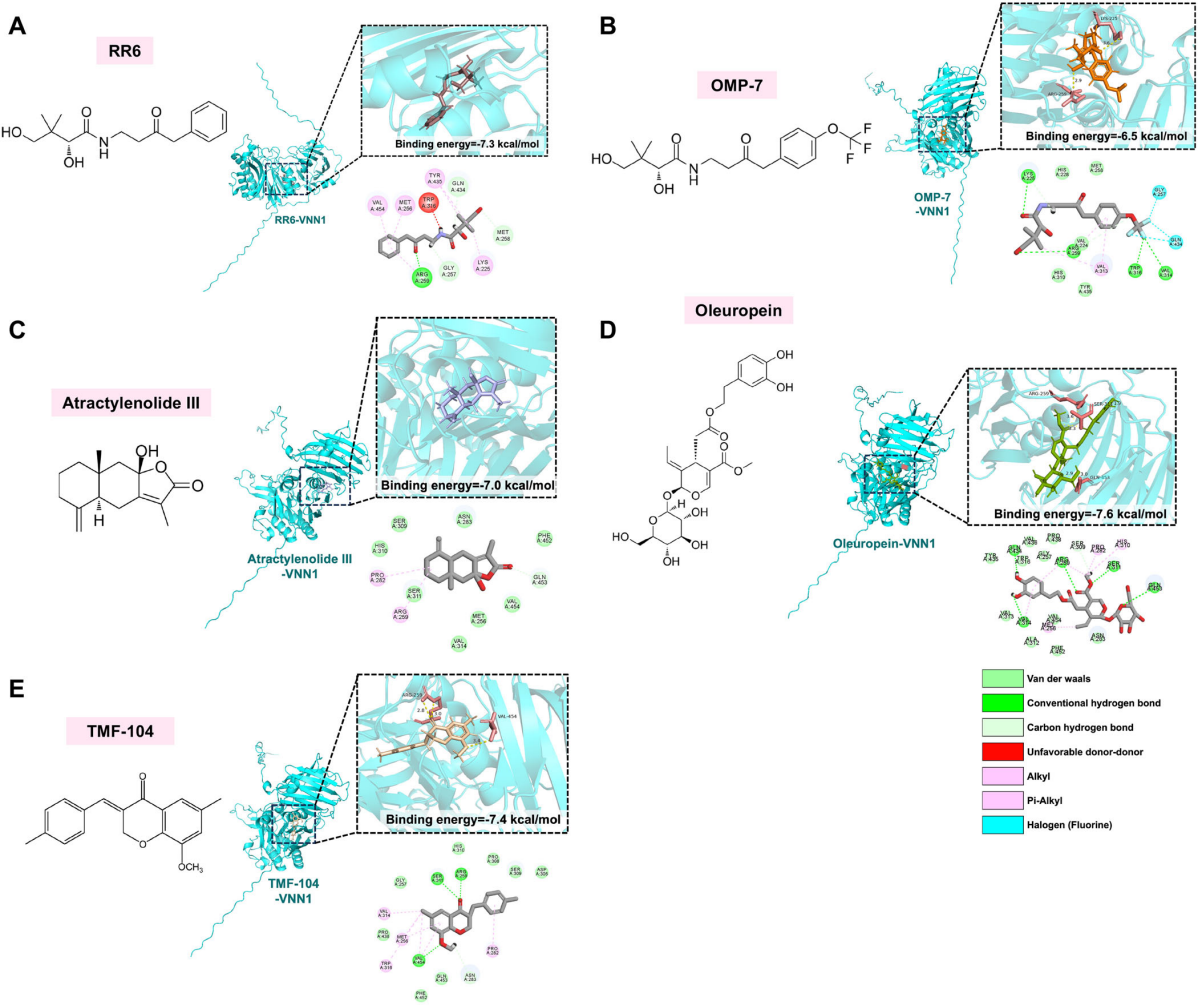
Structural characterization and molecular docking analysis of VNN1 inhibitors. (A–E) Molecular docking simulations were performed between human VNN1 protein and five reported inhibitors using computational modeling approaches. The predicted binding conformations, interaction sites, and corresponding binding energies (kcal/mol) are shown for each ligand‐receptor complex. Structural data of compounds were retrieved from PubChem database. Molecular visualization was conducted using PyMOL (version 3.1) and CB‐Dock2 web server. In the docking diagrams, ligand structures are represented as stick models, and hydrogen bond interactions with key amino acid residues are indicated by dashed lines.

Given the association between VNN1 upregulation and the pathogenesis and progression of various diseases, here we summarize the key characteristics and applications of existing VNN1 inhibitors (Table 2). We further investigate the interactions between human VNN1 and these compounds using molecular docking (Figure 3).

**Table 2:** Characteristics and applications of VNN1 inhibitors.

Name	IC_50_ (nM)	Work concentration	Study model	Study content	Main outcome	Ref.
RR6	540	3 mg/mL drinking water; 50 mg/kg‐single oral dose	Wistar rats (male, 150–200 g)	Plasma FFA, glucose, and cholesterol	VNN1 activity in plasma was chronically/completely suppressed without toxic side effects.	[73]
			Wistar rats, ZDF rats	Obesity, T2DM	Short‐term inhibition of VNN1 activity had no significant effect.	[74]
OMP‐7	38	10 mg/kg‐subcutaneous injection	Normal hamsters	Serum, renal VNN1 activity	Compared with RR6, the lipophilicity and tissue permeability were increased, and the inhibitory effect was stronger.	[75]
ATL‐III	NA	8 mg/kg	BALB/c mice (male 7 weeks old)	Sepsis‐induced ALI	The mRNA and protein levels of VNN1 were inhibited (*p* < 0.05), and the apoptosis rate was significantly reduced.	[52]
Oleuropein	290	NA	DDAV‐based HTS assay	IBD	In vivo data were not available.	[76]
TMF‐104	NA	Cancer cells: GI_50_ dose Bacteria: MIC	MCF‐7, NCI‐H460, Caki‐1, and Vero cells; Gram‐positive bacteria, Gram‐negative bacteria	Antibacterial, anti‐cancer, antioxidant	In vitro dose‐dependent antioxidant; significant activity against Sty, *E. faecalis*, *S. aureus*, and *E. coli*, MIC were 1.5, 2.0, 12.5, and 13.5 μg/mL, respectively.	[54]

Abbreviations: ALI, acute lung injury; *B. cereus*, *Bacillus cereus*; *E. coli*, *Escherichia coli*; *E. faecalis*, *Enterococcus faecalis*; FFA, free fatty acids; MIC, minimum inhibitory concentration; PA, *Pseudomonas aeruginosa*; *S. aureus*, *Staphylococcus aureus*; Sty, *Salmonella typhimurium*; T2DM, Type 2 diabetes mellitus; ZDF, Zucker diabetic fatty.

### 4.1 RR6 and OMP‐7

Currently, RR compounds are the most extensively researched VNN1 inhibitors. In a previous study, researchers replaced the allyl group in RR2 with an aromatic group to create RR6, a potent, selective, reversible, and orally active VNN1 inhibitor with an IC_50_ of 540 nM against recombinant VNN1 [73]. RR6 also inhibited VNNs in human, bovine, and rat serum with IC_50_ values of 40, 41, and 87 nM, respectively [77]. The drug potency of RR6 was evaluated in vivo in animal models of obesity and diabetes [74]. The findings revealed that oral administration of RR6 for 8 days in Zucker diabetic fatty (ZDF) rats effectively inhibited plasma VNN1 activity. However, it did not impact hepatic glucose production, insulin sensitivity, or hepatic steatosis. The findings suggest that for such chronic metabolic diseases, the therapeutic benefits of short‐term broad inhibition of VNN1 may be limited.

Interestingly, RR6 was further optimized, leading to the successful synthesis of 14 novel VNN1 inhibitors, known as the OMP compounds [75]. Among them, OMP‐7, featuring a trifluoromethoxy group at the para position of the benzene ring, exhibited the most potent inhibitory effect, approximately 20 times that of RR6, with an IC_50_ value of 38 nM. In vivo, OMP‐7 demonstrated superior persistence and inhibition of serum and renal VNN1 compared to RR6. Therefore, the potential utility of such compounds is significant. However, further studies are required to comprehensively elucidate the efficacy, safety, and mechanisms of action of these inhibitors, encompassing pharmacokinetics, pharmacodynamics, and potential interactions with other drugs.

### 4.2 MicroRNAs (miRNAs)

miRNA‐122 plays a crucial role in liver development, diseases, and lipid metabolism [78, 79]. Studies indicate that VNN1 is highly expressed in chicken liver [80]. When miRNA‐122 is knocked down in primary chicken hepatocytes, there is a significant increase in VNN1 expression. Further analysis confirmed that overexpressing miRNA‐122 in Chinese hamster ovary cells reduced the expression of a reporter gene containing the chicken VNN1 3′‐UTR, suggesting a direct inhibitory interaction between miRNA‐122 and VNN1. It is noteworthy that VNN1 expression in chickens is also regulated by PPAR‐α and miRNA‐181a‐5, which act as transcriptional activators and negative regulators, respectively.

Recent studies have shown that miRNA‐203 negatively regulates VNN1 [66]. In a mouse model of septic shock, low expression of miRNA‐203 leads to elevated VNN1 levels and inhibition of the AKT signaling pathway. When treated with miRNA‐203 mimic and Vnn1 gene silencing, septic shock mice exhibit reduced apoptosis in lung tissues, lower levels of malondialdehyde (MDA), alanine aminotransferase (ALT), and aspartate aminotransferase (AST), decreased serum levels of TNF‐α, IL‐1β, IFN‐γ, IL‐10, and IL‐6, reduced polymorphonuclear neutrophils (PMNs) and pulmonary alveolar macrophages (PAMs) in bronchoalveolar lavage fluid (BALF), and increased superoxide dismutase (SOD) activity. In the future, miRNA‐targeted inhibition of VNN1 to alleviate inflammation could be a promising direction for exploration.

### 4.3 Compounds of Natural Origin

Small molecule compounds derived from natural plants have become a highly popular research area in medicine. Atractylenolide III (ATL‐III), a bioactive compound from *Atractylodes macrocephala* Koidz, exhibits numerous beneficial effects, including antioxidant, anti‐tumor, anti‐allergic, anti‐bacterial, and cognitive protective properties [81, 82]. Additionally, ATL‐III has been shown to reduce pulmonary fibrosis and significantly improve lung function through multiple pathways [83, 84]. It also alleviates sepsis‐induced lung injury by inhibiting VNN1 protein expression [52]. Studies have demonstrated that ATL‐III administration (Bohu Biotechnology Co., Shanghai, China) reduces sepsis‐induced lung injury and apoptosis and inhibits the secretion of inflammatory factors, primarily TNF‐α, IL‐1β, and IL‐6. Mechanistically, ATL‐III upregulates Bcl‐2 expression while reducing Bax, caspase‐3, VNN1, and Forkhead Box protein O1 (FoxO1) expression.

Clinical trials have shown that extra virgin olive oil consumption can lower the risk of several diseases [85, 86]. Oleuropein (3,4‐dihydroxyphenylethanol), an ester of elenolic acid and hydroxytyrosol found in green olives and leaves, exhibits antioxidant properties by directly inhibiting PPAR‐γ transcriptional activity [87, 88]. Furthermore, oleuropein induces apoptosis in cancer cells through a p53‐dependent pathway and regulation of Bax and Bcl‐2 genes [86]. Tian et al. [76] developed a novel enzyme‐activated near‐infrared (NIR) fluorescent probe, DDAV, with high selectivity and sensitivity towards VNN1. They also established a high‐throughput screening (HTS) method based on DDAV. By screening 92 herbal medicines, they identified oleuropein as a potential natural inhibitor of VNN1 (IC_50_ = 290 nM). This finding provides a new approach for screening compounds derived from natural sources.

### 4.4 Others

Researchers have developed a highly sensitive fluorescence assay and HTS method using labeled pantothenic acid derivatives. This includes a fluorescent substrate called pantothenate‐AMC (pantothenate‐7‐amino‐4‐methylcoumarin), significantly enhancing HTS sensitivity [77, 89]. Additionally, pyrimidinamide compounds have been synthesized as core structures for VNN inhibitors [90]. These inhibitors are coupled with probes to evaluate their efficacy and monitor VNN1 activity. In vivo experiments highlight the clinical promise of VNN1 inhibitors in the treatment of inflammatory diseases [22]. Furthermore, Saif et al. [54] reported a novel benzylidene chromanone compound, 3‐benzylidene chroman‐4‐one derivative (TMF‐104), with a strong binding affinity to VNN1 protein. In vitro experiments showed significant dose‐dependent antioxidant and antibacterial activities, along with inhibition of cancer cell proliferation and pro‐apoptotic effects (see Table 2).

Lipolysis is a critical metabolic process that regulates fat content and maintains energy balance in adipose tissue. Dysregulation of lipolysis can lead to excessive fat accumulation and contribute to metabolic disorders, such as obesity, diabetes, and hyperlipidemia [91, 92]. A nanosystem composed of a P3‐peptide, chitosan oligosaccharide lactate, and polyethylene glycol, targeting VNN1 expression in abdominal white adipose tissue (WAT), successfully restored impaired lipolysis and improved glucose/insulin intolerance in diabetic db/db mice [93]. This indicates that targeting VNN1 can regulate signaling pathways and lipid metabolism without affecting the production or secretion of insulin, which can be considered a promising idea for drug delivery carriers.

## 5 Conclusions and Future Perspectives

At the nexus of inflammation and metabolism, VNN1 assumes a central regulatory role. Metabolites generated via the VNN1‐pantetheinase pathway—pantothenic acid and cysteamine—are essential for meeting the metabolic demands of cells. Its functions exhibit marked context‐dependency across acute inflammation, trauma, chronic diseases, and cancer, intricately intertwined with the metabolic landscapes of specific cells and tissues. In the context of acute inflammation and trauma, VNN1 exerts its influence predominantly within immune cells, including lymphocytes and macrophages. Studies utilizing VNN1‐deficient murine models have demonstrated attenuated disease phenotypes, underscoring VNN1′s critical function in orchestrating both metabolic and inflammatory processes.

VNN1′s mode of action diverges significantly between acute and chronic pathologies. During acute inflammatory episodes, VNN1 rapidly mobilizes immune responses; however, unchecked activation can lead to dysregulated, excessive inflammation. In contrast, in chronic diseases, persistent VNN1 activity within tissues harboring activated myofibroblasts sustains inflammatory signaling. Concurrently, metabolites of the VNN1‐pantetheinase pathway maintain cellular metabolic homeostasis by regulating CoA turnover and the GSH/GSSG redox balance.

In the tumor microenvironment, VNN1 promotes inflammation and impacts tumor cell survival pathways. High VNN1 expression in tumor associated tissues creates a niche for immune evasion, invasion, and metastasis, though its oncogenic mechanisms remain unclear.

Several VNN1 inhibitors, including RR6, OMP‐7, oleuropein, and ATL‐III, have been demonstrated to possess anti‐inflammatory, antioxidant, and antibacterial potential. However, pharmacological inhibition or genetic deletion of VNN1 may transiently disrupt the metabolic integrity of the mucosal barrier, which poses significant challenges for clinical translation. Molecular docking studies reveal that naturally derived compounds such as oleuropein and ATL‐III form stable interactions with human VNN1 through multiple amino acid residues, undoubtedly representing a direction worthy of exploration. Future research efforts should prioritize the development of selective VNN1 inhibitors, regulation of downstream signaling pathways, and discovery of bioactive natural compounds. Combining these novel therapeutic approaches with existing diagnostic and treatment strategies holds promise for the development of next‐generation anti‐inflammatory and antioxidant therapies, although extensive preclinical and clinical validation is still required.

## Author Contributions

**Linxi Lv:** conceptualization, writing – original draft, writing – review and editing. **Tian Wang:** visualization, writing – review and editing, writing – original draft. **Wenzhan Xie:** writing – review and editing. **Jialong Wei:** writing – review and editing. **Laixian Zhou:** writing – review and editing. **Xiaopei Qiu:** writing – review and editing. **Hui Feng:** writing – review and editing, conceptualization, supervision, writing – original draft. **Wei Gu:** conceptualization, writing – original draft, writing – review and editing, supervision.

## Conflicts of Interest

The authors declare no conflicts of interest.

## Data Availability

Data sharing is not applicable to this article as no new data were created or analyzed in this study. No data was used for the research described in the article.
